# Supplementary material for: Exploring the Molecular Players behind the Potentiation of Chemotherapy Effects by Durvalumab in Lung Adenocarcinoma Cell Lines
Source: Pharmaceutics. 2023 May 12;15(5):1485. doi: 10.3390/pharmaceutics15051485 (PMC10221650; doi:10.3390/pharmaceutics15051485)
Supplement: Supplementary file 1 [file pharmaceutics-15-01485-s001.zip › pharmaceutics-2306971_Supplementary Figures and Table legends.pdf]

SUPPLEMENTARY LEGENDS

Exploring the molecular players behind the potentiation of chemotherapy effects by durvalumab in lung adenocarcinoma cell lines

Marika Saar [1,2,3], Jana Jaal [1,4], Alvin Meltsov [5,6], Tõnis Laasfeld [7,8], Helen Lust [1], Sergo Kasvandik [9] and Darja Lavogina[1,5,7,*]

[1]Institute of Clinical Medicine, Faculty of Medicine, University of Tartu, 50406 Tartu, Estonia

[2]Institute of Pharmacy, University of Tartu, 50411 Tartu, Estonia

[3]Pharmacy, Tartu University Hospital, 50406 Tartu, Estonia

[4]Haematology and Oncology Clinic, Tartu University Hospital, 50406 Tartu, Estonia

[5]Competence Centre on Health Technologies, 50411 Tartu, Estonia

[6]Department of Genetics and Cell Biology, GROW School for Oncology and Developmental Biology, Maastricht University, 6200 MD Maastricht, The Netherlands

[7]Institute of Chemistry, University of Tartu, 50411 Tartu, Estonia

[8]Department of Computer Science, University of Tartu, 51009 Tartu, Estonia

[9]Proteomics Core Facility, Institute of Technology, University of Tartu, 50411 Tartu, Estonia

*Correspondence: darja.lavogina@ut.ee; Tel. +372-737-5296

Supplementary Table S1. Lists of proteins with significantly different abundances (FDR < 0.1) in A549 samples treated with individual drugs versus drug mixtures (N = 3)

- Part A: cells treated with cisplatin or mixture of cisplatin with durvalumab
- Part B: cells treated with durvalumab or mixture of cisplatin with durvalumab
- Part C: cells treated with pemetrexed or mixture of pemetrexed with durvalumab
- Part D: cells treated with durvalumab or mixture of pemetrexed with durvalumab

Abbreviations: log_2FC, binary logarithm of fold change (positive numbers indicate higher abundance in the mixture-treated cells); MIX1, mixture of cisplatin with durvalumab; MIX2, mixture of pemetrexed with durvalumab. Proteins for which the FDR value is greater than 0.05 are shown in orange; green highlight indicates positive log_2FC values and blue highlight indicates negative log_2FC values.

Supplementary Table S2. Lists of proteins with significantly different abundances (FDR < 0.1) in A549 samples treated with individual drugs versus drug mixtures (N = 3)

- Part A: cells treated with cisplatin or mixture of cisplatin with durvalumab
- Part B: cells treated with durvalumab or mixture of cisplatin with durvalumab
- Part C: cells treated with pemetrexed or mixture of pemetrexed with durvalumab
- Part D: cells treated with durvalumab or mixture of pemetrexed with durvalumab

Abbreviations: log_2FC, binary logarithm of fold change (positive numbers indicate higher abundance in the mixture-treated cells); MIX1, mixture of cisplatin with durvalumab; MIX2, mixture of pemetrexed with durvalumab. Proteins for which the FDR value is greater than 0.05 are shown in orange; green highlight indicates positive log_2FC values and blue highlight indicates negative log_2FC values.

Supplementary Table S3. Lists of proteins with commonly altered expression levels in different treatment comparisons within the same line or in comparison of the same treatment between the cell lines

The identical proteins (shown as red text with red highlight) were found by comparing the lists presented in the Supplementary Table S1 and S2, and correspond to the overlapping areas of the Venn diagram presented in Figure 4 of the main text.

- Part I. Effect of chemotherapeutic agent addition – commonly altered proteins in the following comparisons
    - MIX1 *vs* D in A549 and HCC-44
    - MIX2 *vs* D in A549 and HCC-44
    - MIX1 *vs* D and MIX2 *vs* D in A549
    - MIX1 *vs* D and MIX2 *vs* D in HCC-44
- Part II. Effect of durvalumab addition – commonly altered proteins in the following comparisons:
    - MIX1 *vs* C in A549 and HCC-44
    - MIX2 *vs* P in A549 and HCC-44
    - MIX1 *vs* C and MIX2 *vs* P in A549
    - MIX1 *vs* C and MIX2 *vs* P in HCC-44

Abbreviations: MIX1, mixture of cisplatin with durvalumab; MIX2, mixture of pemetrexed with durvalumab.

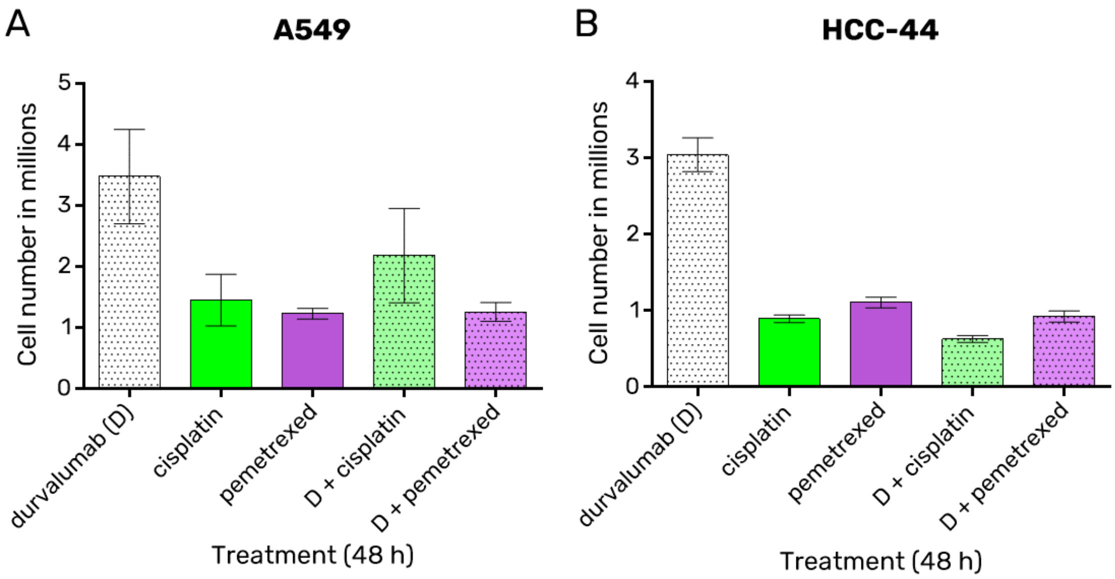

Supplementary Figure S1. Cell counts in treated samples prior to pelleting and freezing

Number of cells established after 48-h treatment prior to the proteomics experiments. Each graph shows mean +/- standard error of mean (N = 3). (A) A549 cell line, (B) HCC-44 cell line. According to the previously published data (https://doi.org/10.3892/ol.2023.13738), durvalumab alone does not significantly affect the cell viability.

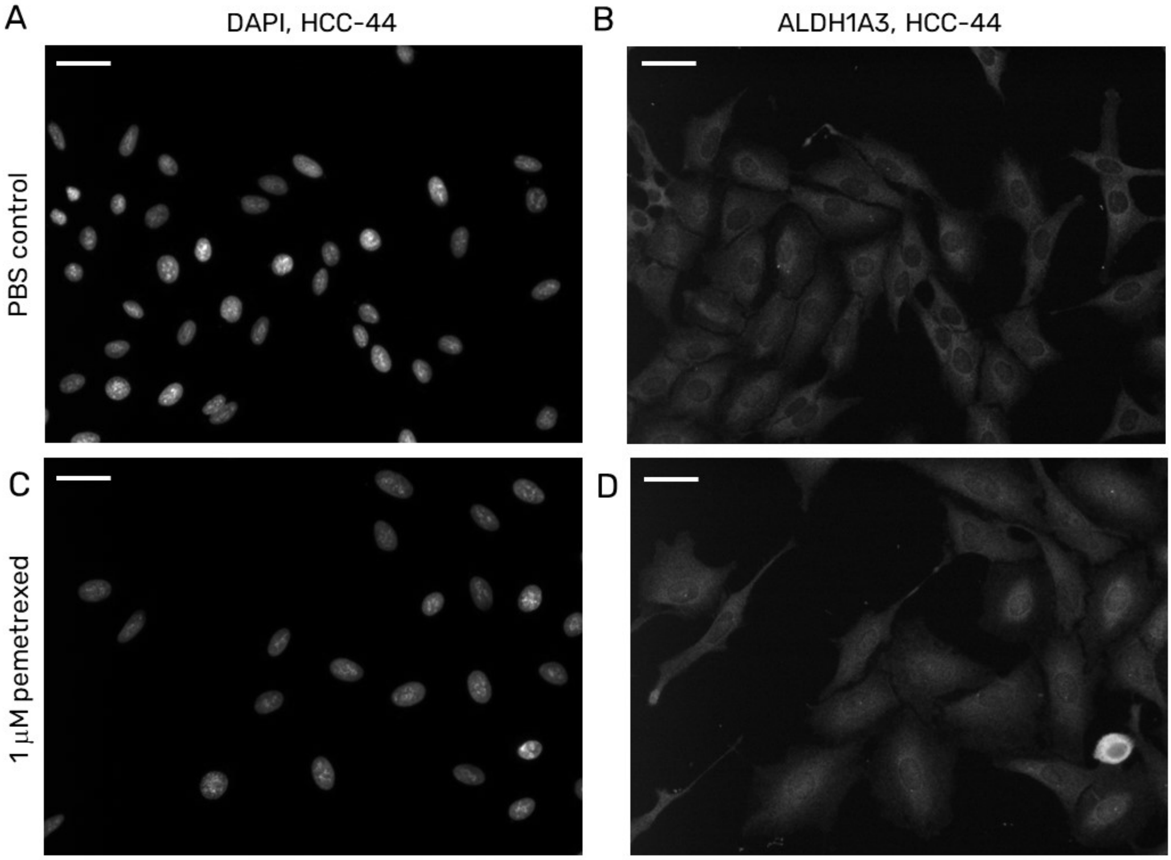

Supplementary Figure S2. Microscopy images showing the immunostaining of aldehyde dehydrogenase 1 family member A3 (ALDH1A3) and nuclear staining (DAPI) in HCC-44 cell line

The treatment conditions are listed on the left and the imaging channels at the top. The images were taken with the 20× objective; scale bar (top left): 50 μM. For better visualization, the brightness in ALDH1A3 channel was enhanced by 40% (the quantification was performed with unmodified data).

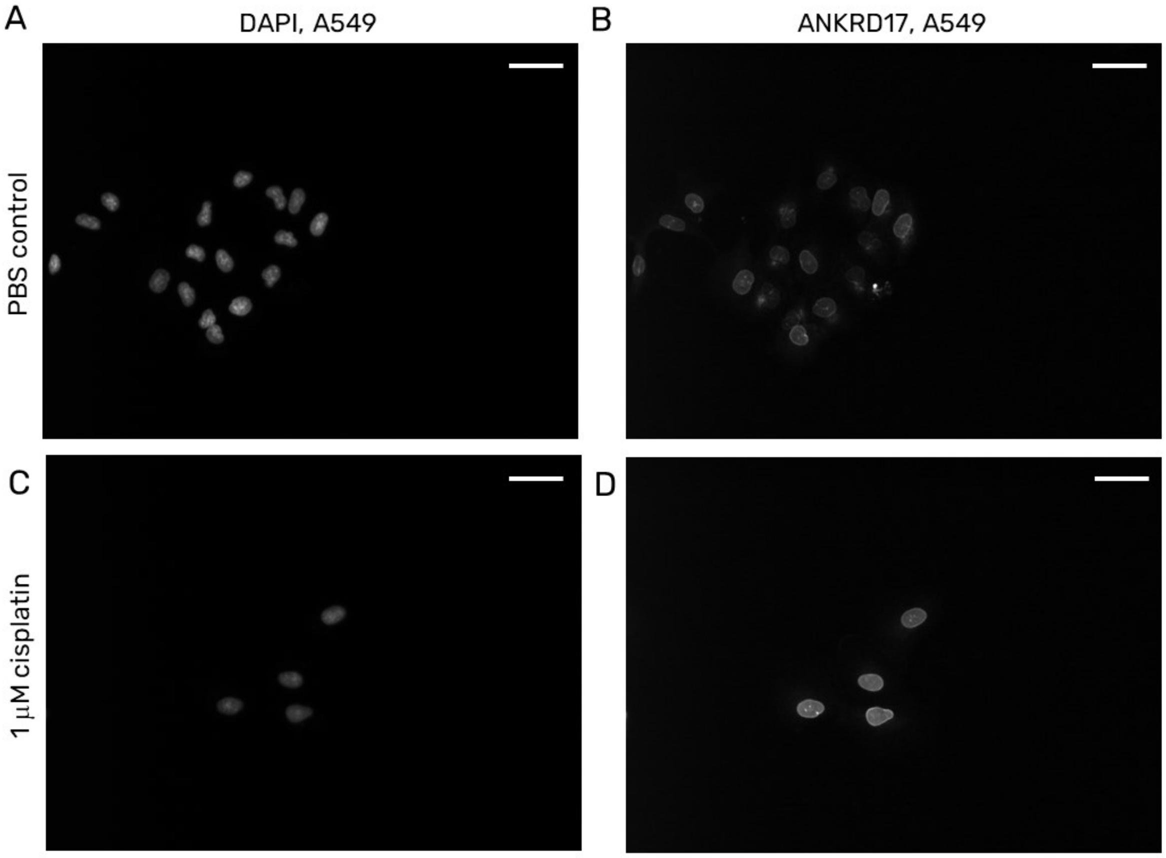

Supplementary Figure S3. Microscopy images showing the immunostaining of ankyrin repeat domain-containing protein 17 (ANKRD17) and nuclear staining (DAPI) in A549 cell line

The treatment conditions are listed on the left and the imaging channels at the top. The images were taken with the 20× objective; scale bar (top right): 50 μM.

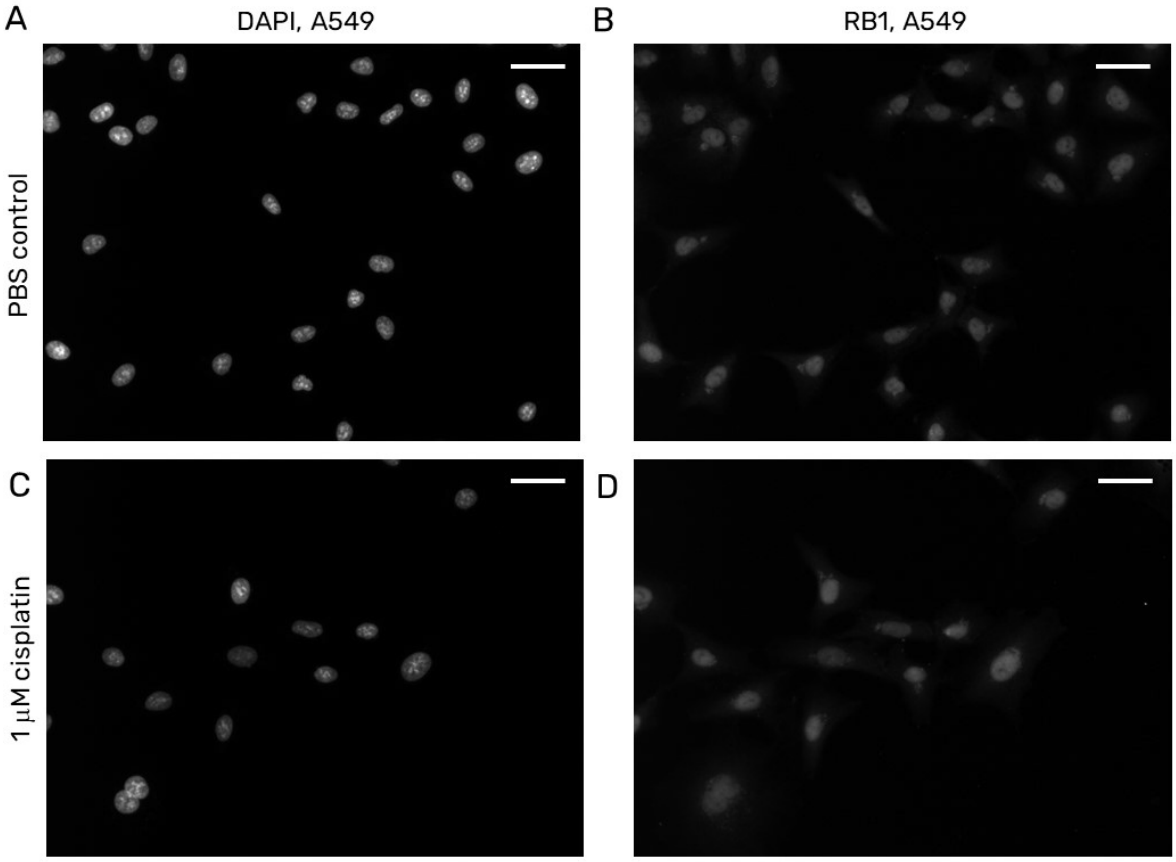

Supplementary Figure S4. Microscopy images showing the immunostaining of retinoblastoma transcriptional corepressor 1 (RB1) and nuclear staining (DAPI) in A549 cell line

The treatment conditions are listed on the left and the imaging channels at the top. The images were taken with the 20× objective; scale bar (top right): 50 μM.

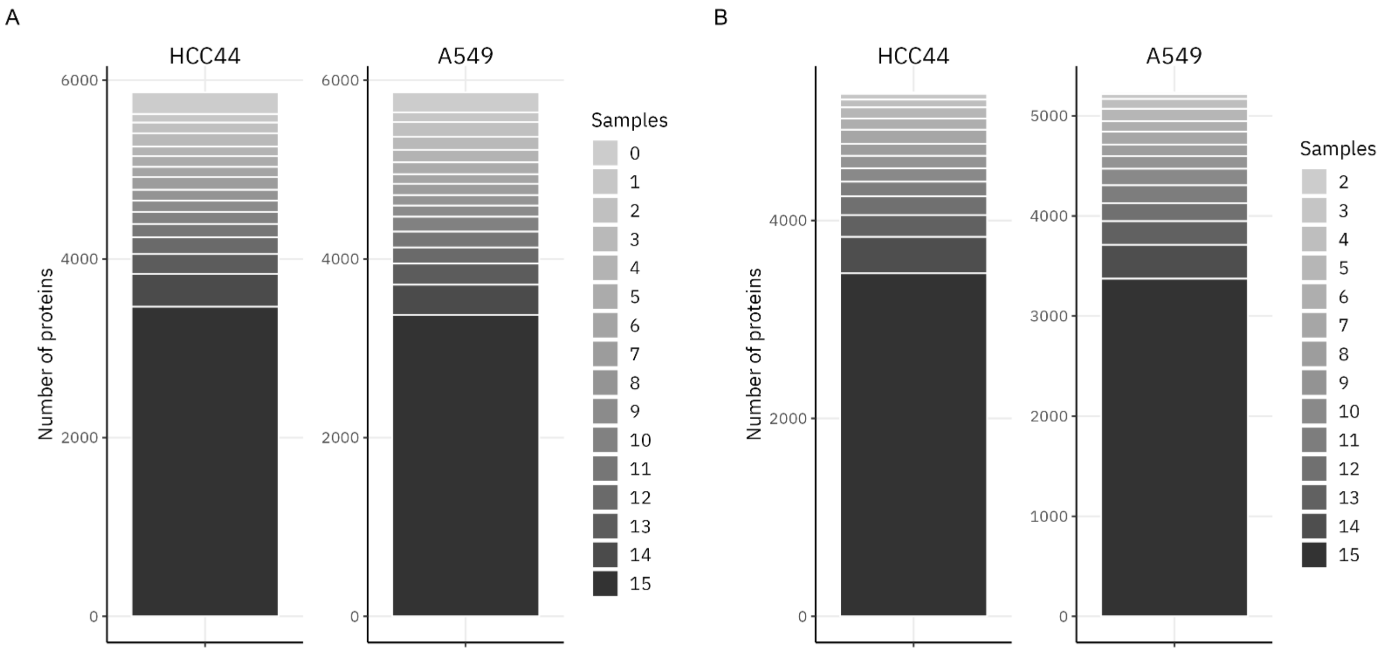

Supplementary Figure S5. Protein coverage across the number of samples in HCC-44 and A549 cell lines

Coverage diagram showing in how many samples a protein was expressed before (A) and after filtering out (B) the genes that are expressed in at least two replicates in any treatment.

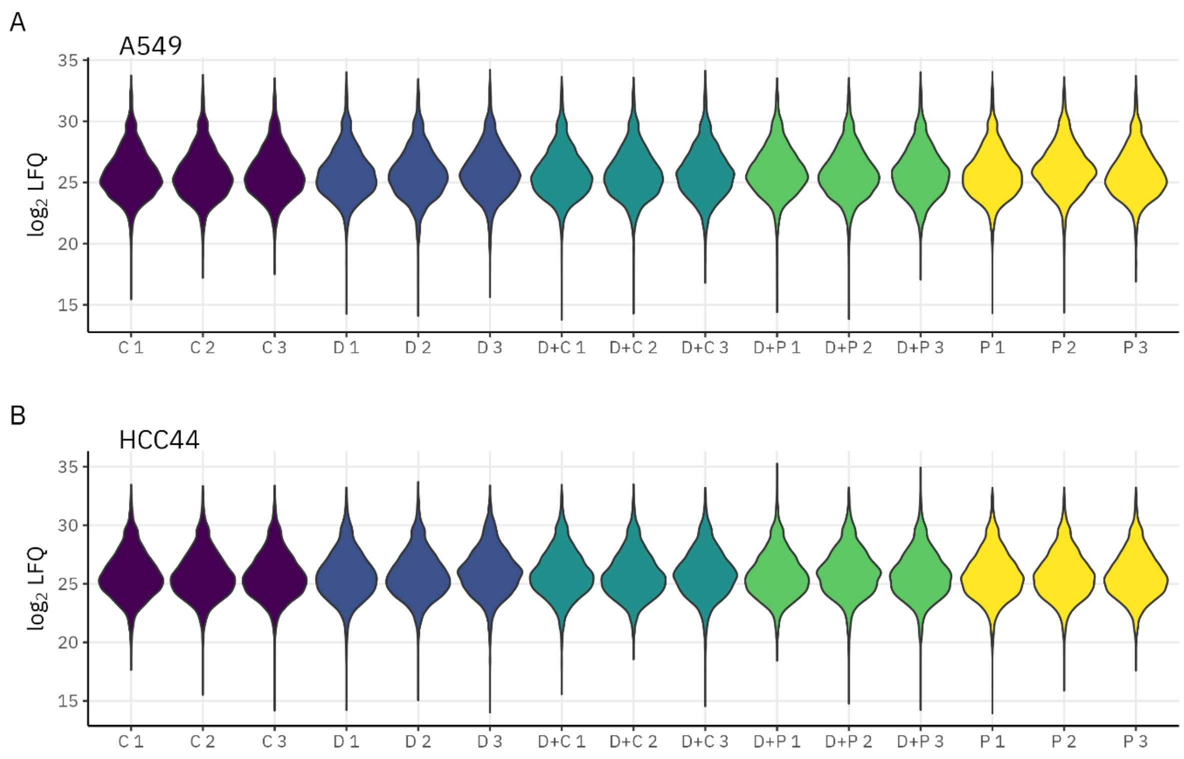

Supplementary Figure S6. Distribution of protein counts in differently treated samples

(A) A549 cell line, (B) HCC-44 cell line. Counts were logarithm-transformed and distributions are estimated using a Gaussian kernel.

Abbreviations: C stands for cisplatin, D for durvalumab, D+C for durvalumab and cisplatin mixture, D+P for durvalumab and pemetrexed mixture, LFQ for normalized label-free quantification intensity, P for pemetrexed. The numbers indicate independent experiments.

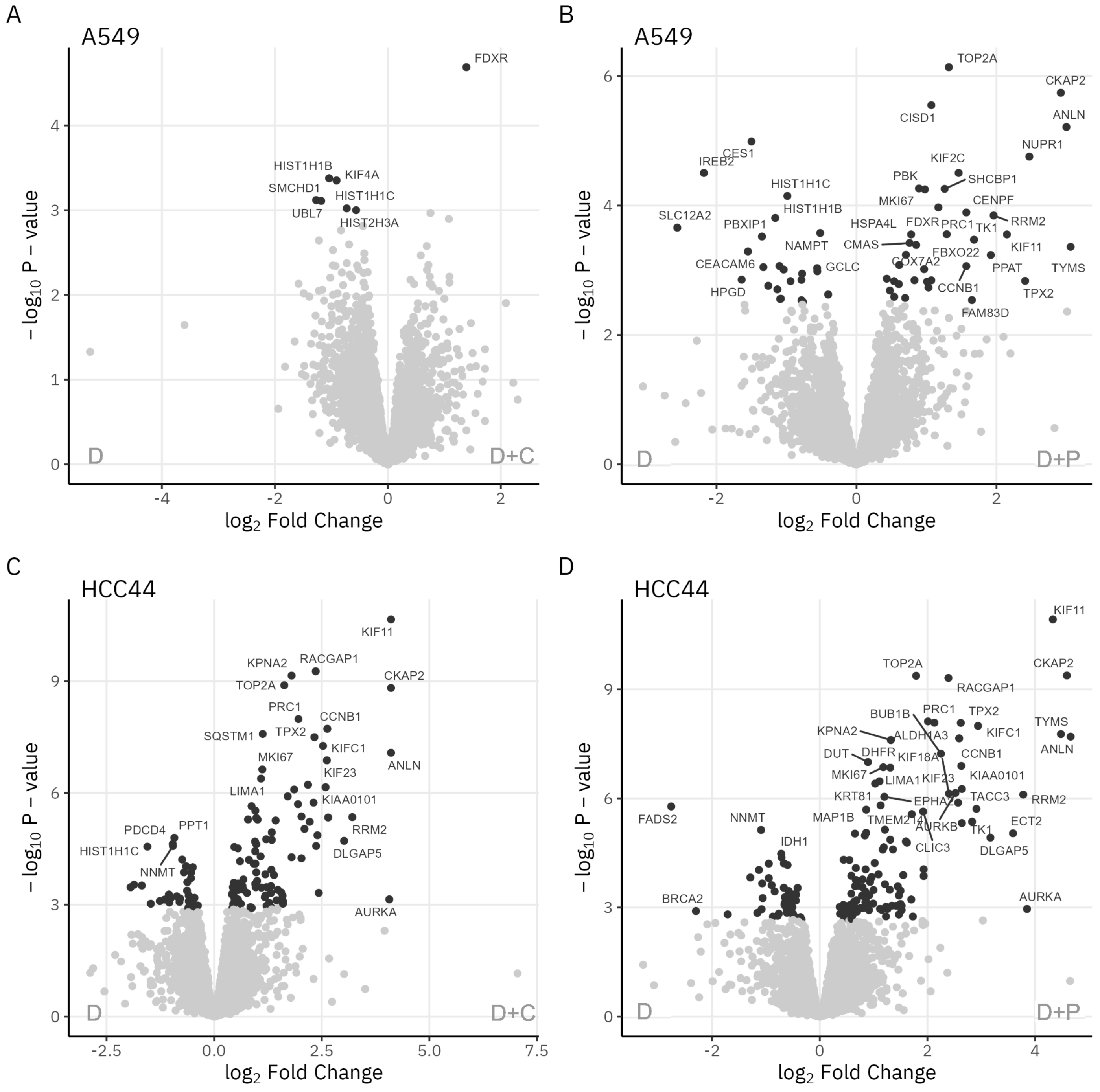

Supplementary Figure S7. Volcano plots showing effect of chemotherapeutic agent addition to the treatment mixture

Proteins marked in black are considered significantly differentially expressed (FDR < 0.05).

A-B: A549 cell line, comparison of treatments D+C *vs* D (A) and D+P *vs* D (B);

C-D: HCC-44 cell line, comparison of treatments D+C *vs* D (C) and D+P *vs* D (D).

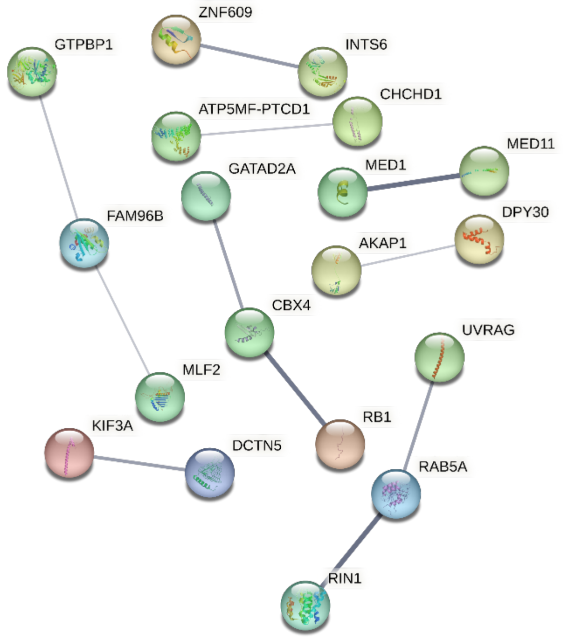

Supplementary Figure S8. Cellular networks identified by the STRING algorithm based on the differential abundances of proteins in A549 cells treated with cisplatin versus *cisplatin and durvalumab mixture (proteomics FDR < 0.1)*

Here and below, grey line thickness between the bubbles indicates the strength of data support.

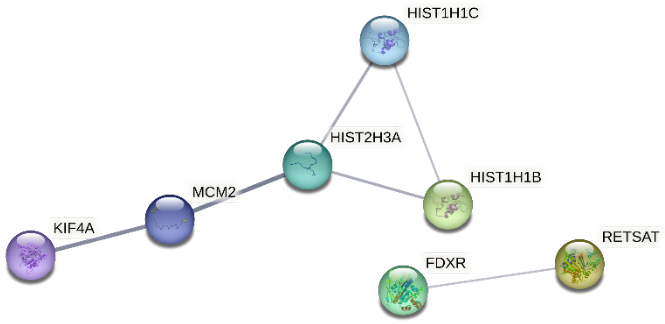

Supplementary Figure S9. Cellular networks identified by the STRING algorithm based on the differential abundances of proteins in A549 cells treated with durvalumab versus *cisplatin and durvalumab mixture (proteomics FDR < 0.1)*

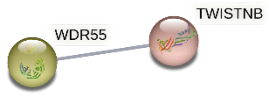

Supplementary Figure S10. Cellular network identified by the STRING algorithm based on the differential abundances of proteins in A549 cells treated with pemetrexed versus *pemetrexed and durvalumab mixture (proteomics FDR < 0.1)*

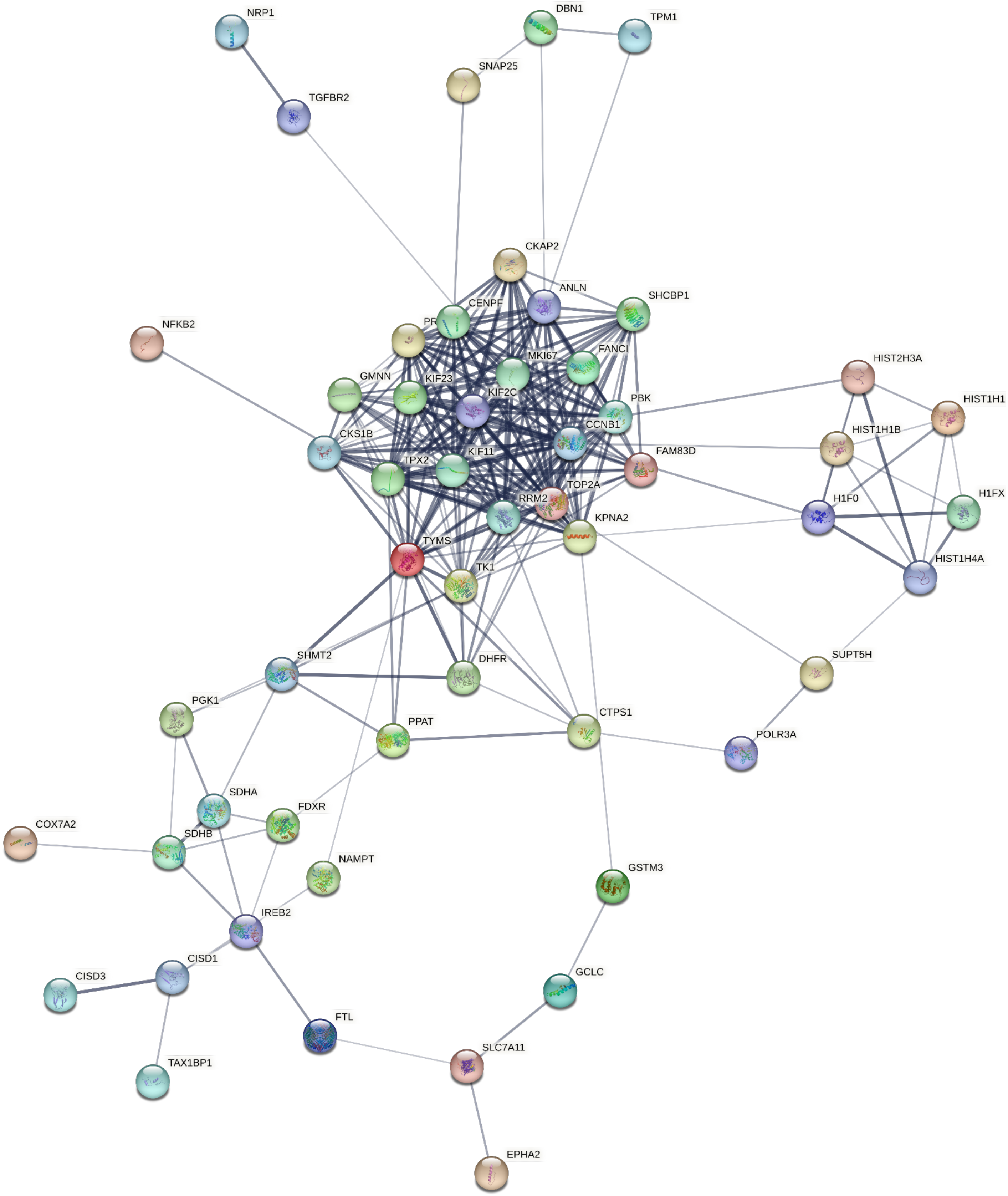

Supplementary Figure S11. Cellular networks identified by the STRING algorithm based on the differential abundances of proteins in A549 cells treated with durvalumab versus *pemetrexed and durvalumab mixture (proteomics FDR < 0.1)*

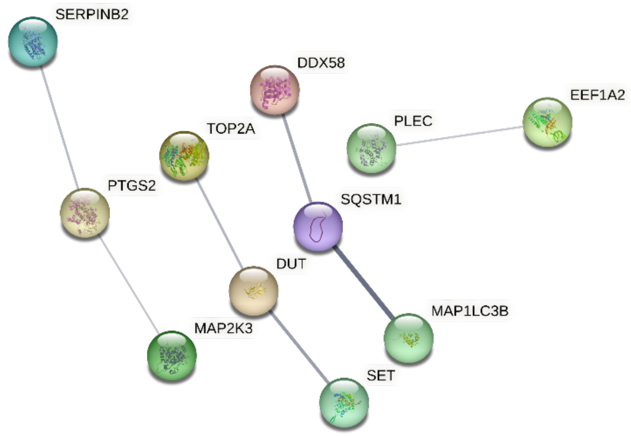

Supplementary Figure S12. Cellular networks identified by the STRING algorithm based on the differential abundances of proteins in HCC-44 cells treated with cisplatin versus *cisplatin and durvalumab mixture (proteomics FDR < 0.1)*

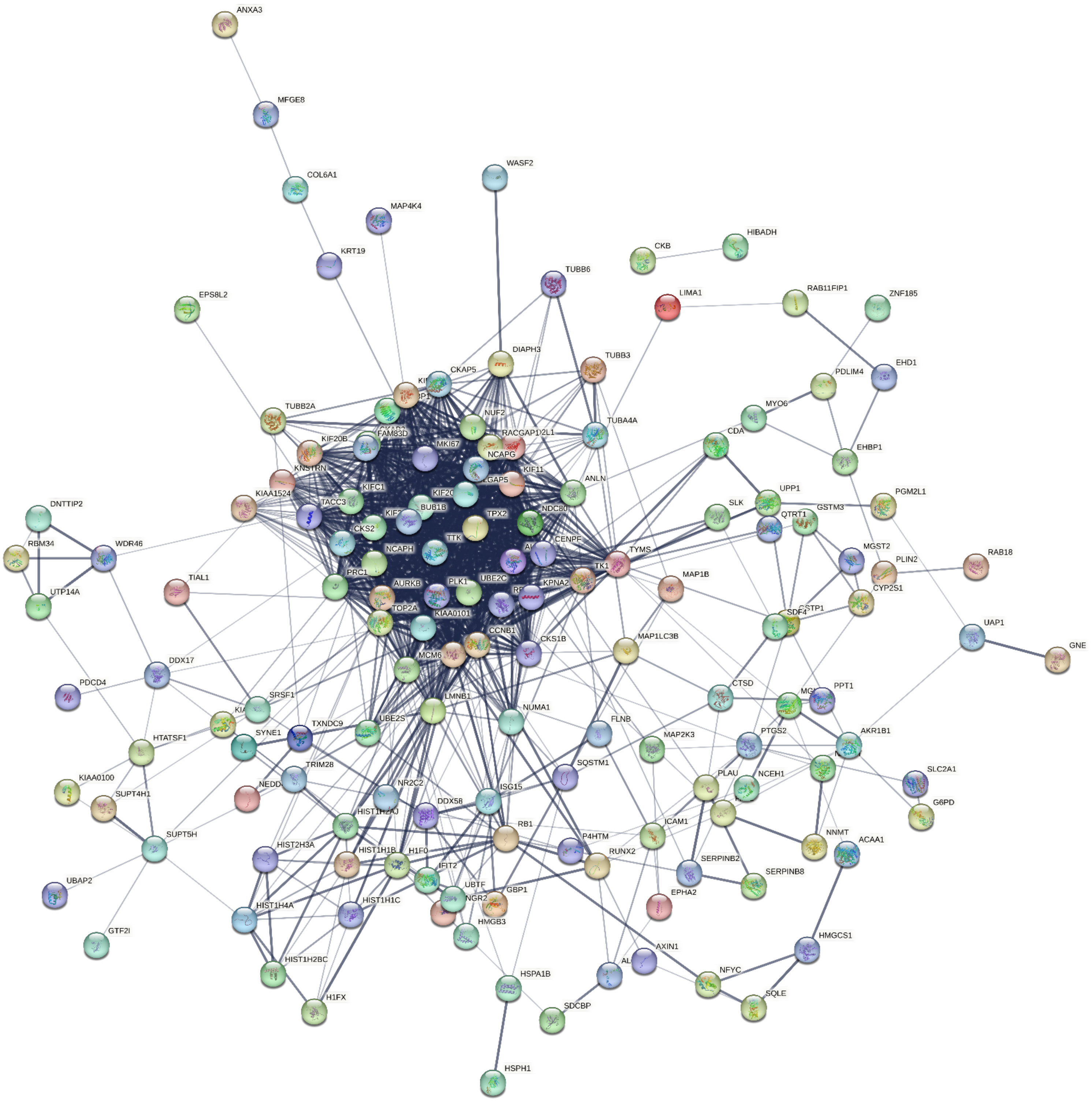

Supplementary Figure S13. Cellular networks identified by the STRING algorithm based on the differential abundances of proteins in HCC-44 cells treated with durvalumab versus *cisplatin and durvalumab mixture (proteomics FDR < 0.1)*

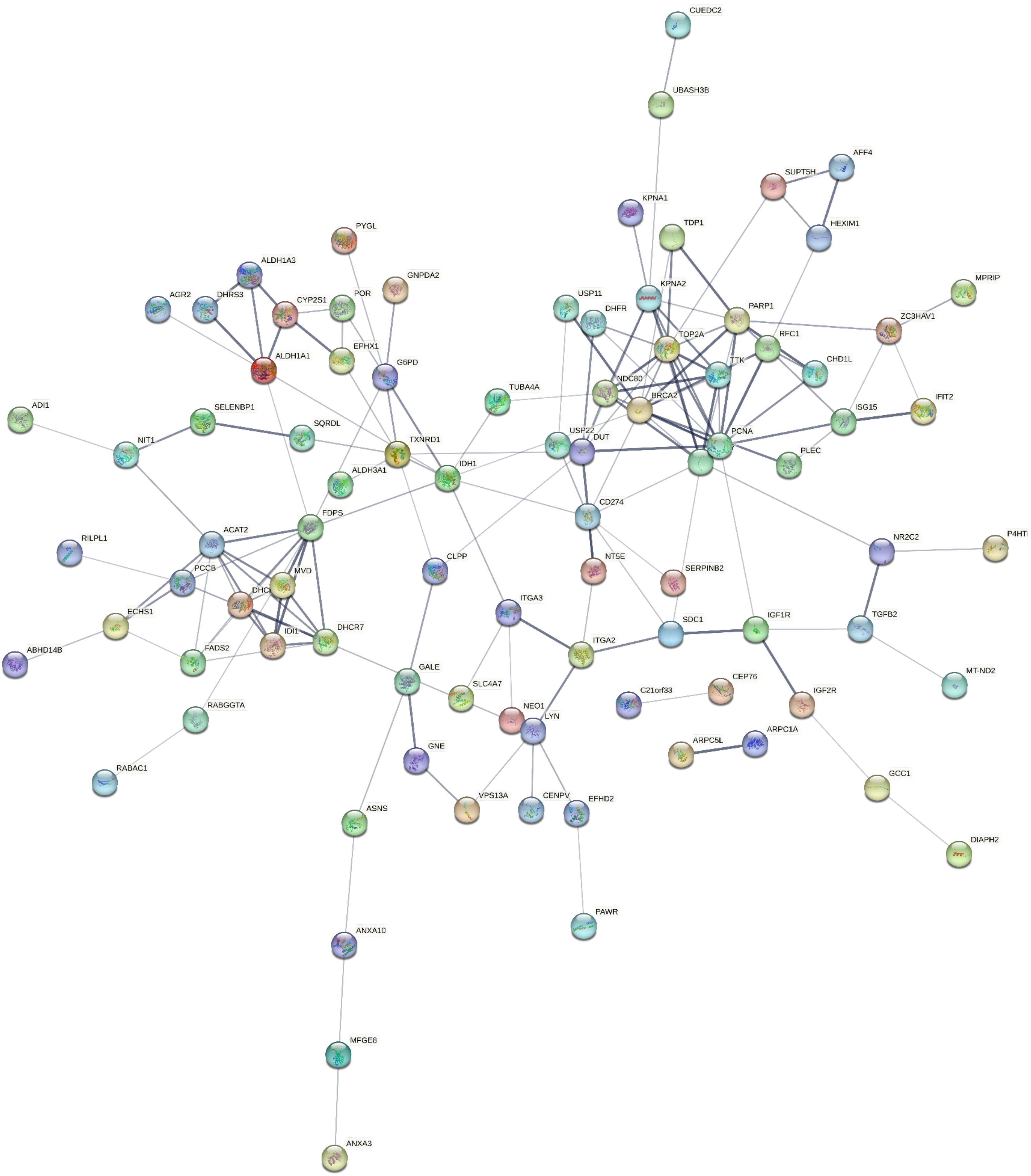

Supplementary Figure S14. Cellular networks identified by the STRING algorithm based on the differential abundances of proteins in HCC-44 cells treated with pemetrexed versus *pemetrexed and durvalumab mixture (proteomics FDR < 0.1)*

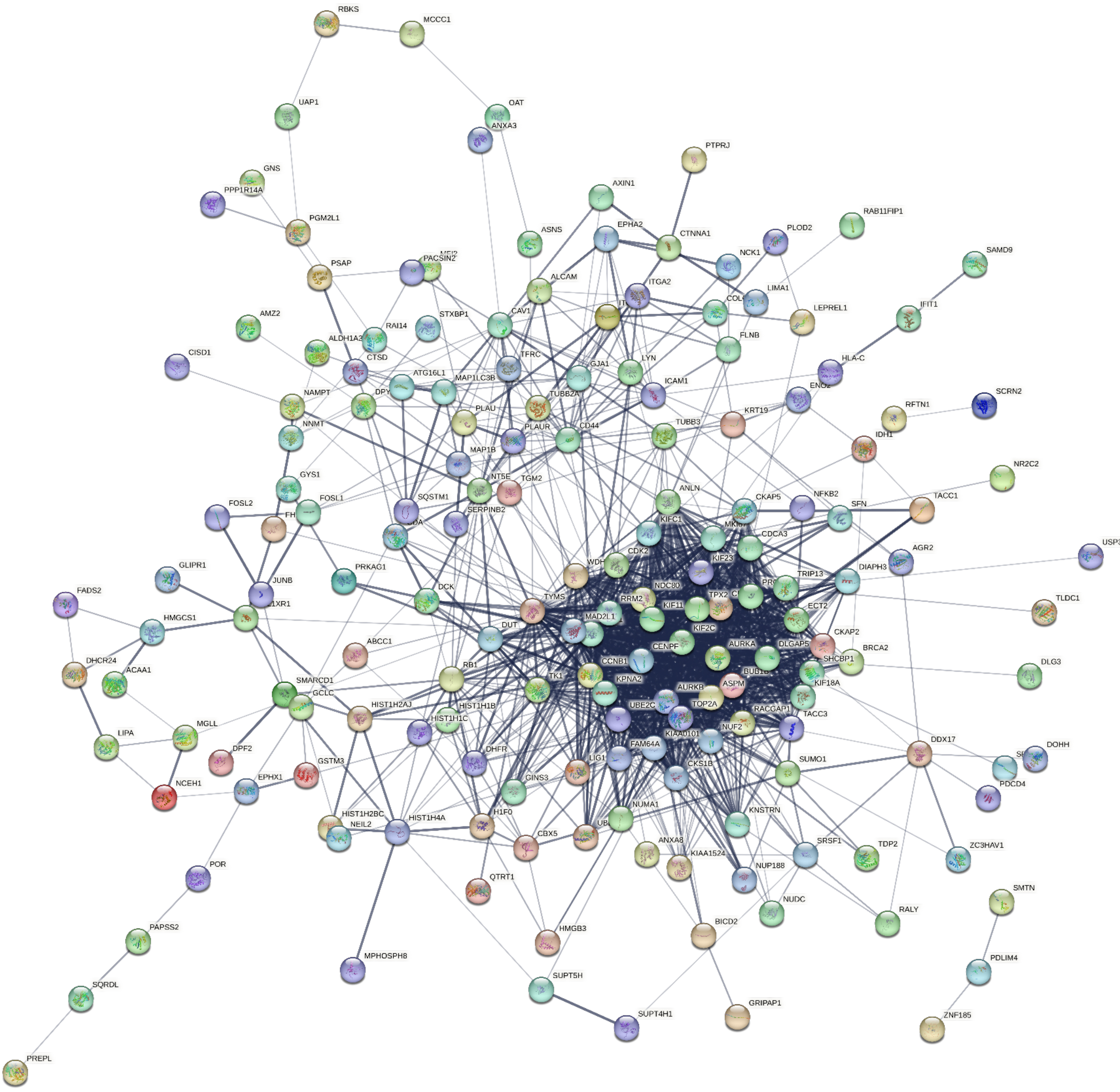

Supplementary Figure S15. Cellular networks identified by the STRING algorithm based on the differential abundances of proteins in HCC-44 cells treated with durvalumab versus *pemetrexed and durvalumab mixture (proteomics FDR < 0.1)*
